# Assessment of snakebite management practices at Meserani Juu in Monduli District, Northern Tanzania

**DOI:** 10.1371/journal.pone.0278940

**Published:** 2022-12-22

**Authors:** Shabani Iddi, Joseph Justin, Kayo Hamasaki, Eveline T. Konje, Gilbert W. Kongola

**Affiliations:** 1 Department of Physiology, Weill Bugando School of Medicine, Catholic University of Health and Allied Sciences, Mwanza, Tanzania; 2 School of Pharmacy, Catholic University of Health and Allied Sciences, Mwanza, Tanzania; 3 Department of Biostatistics and Epidemiology, School of Public Health, Catholic University of Health and Allied Sciences, Mwanza, Tanzania; Instituto Butantan, BRAZIL

## Abstract

**Background:**

Snakebite envenoming represents a tragically neglected tropical disease mostly affecting poor people living in remote areas of developing countries, primarily in sub-Saharan Africa. Anti-snake venom (ASV) is the only approved specific treatment for systemic envenoming from snakebite, but it remains largely unavailable in many parts of developing countries. There is paucity of data on snakebite management practice in Tanzania. This study aimed at assessing the community management practices of snakebite and availability of anti-snake venom in the public health facilities in Monduli District, Northern Tanzania.

**Methods:**

A cross sectional study was carried out between May and June, 2018 involving 67 victims, 147 other household members, and 35 public health facilities. A structured questionnaire, respondent interview, and health facility report/document review were considered during data collection. Clean data were analyzed using SPSS version 20.

**Results:**

Sixty-seven snakebite victims and 147 other household members were interviewed during a household survey. All snakebite cases reported to having visited a health facility after snakebite with the majority 55/67 (82.1%) reporting the use, prior attendance to medical care, of some form of local treatment such as tourniquets 13 (19.4%), local incision 11 (16.4%), and snakestone 7 (10.4%). None of the public health facilities in Monduli District attended a snakebite case and had never stocked anti-snake venom products. In this area, 45 snakebite cases were reported to be managed at Meserani snake park clinic where anti-snake venom products were available and provided for free in the period between January 2017 and December 2017.

**Conclusion:**

Majority of the snakebite cases at Meserani Juu relied on local methods for the management of snake bites of which most are of unknown efficacy and safety. Furthermore, none of the primary public health facilities in Monduli District stocked antivenom despite being a habitat for different kinds of venomous snakes. The government and local non-government organizations should collaborate so as to improve the anti-snake venom availability and the provision of snakebite preventive and management awareness programs, especially to the rural communities.

## Background

Snakebite envenoming represents a significant public health problem in many developing countries, especially in rural tropical regions of the world, affecting mostly agriculturalists and pastoralists and having a serious impact in terms of morbidity and mortality [1–3]. The estimated number of snakebites in the world ranges from 550,000 to 1,200,000 million individuals [1], but the figure may reach up to 5,400,000 [4]. Among those individuals, 421,000 to 1,841,000 are envenomed, and more than 81,000 die every year [1, 4, 5]. In Africa, about 1,000,000 snakebites occur every year, primarily in Sub-Saharan Africa, resulting in 100,000 to 500,000 cases of envenoming, and at least 10,000 to 30,000 deaths [4, 6]. A more recent study in West Africa have reported the annual snake bite mortality ranged from 24 (95% confidence interval: 19–29) in Guinea-Bissau to 1927 (1529–2333) in Nigeria. In the same study, amputations ranged from 28 (17–48) in Guinea-Bissau with highest estimates of 2368 (1506–4043) in Nigeria [7]. In west Africa, snake bite envenoming is a public health concern similarly to other widely recognized neglected tropical diseases such as Echinococcosis, intestinal nematode infections, Leishmaniasis, Onchocerchiasis, Trachoma and Trypanosomiasis [5, 7].

It is difficult to estimate the true incidence rate of snakebites envenomation in rural tropical and sub-tropical regions, particularly in Sub-Saharan Africa where the majority of the snakebites cases are undocumented [1, 6, 8]. This is mainly due to the failure of many victims to attend health facilities and rely- instead on local traditional healers or the use of some forms of local traditional methods which have not been medically or scientifically proven to be efficacious in the management of snakebite victims [9–14], a fact that further complicates this problem [15]. Traditional methods of treatment have been associated with delay in seeking proper treatment causing many deaths to occur before they reach the hospital [6].

Anti-snake venom (ASV) is the only approved specific treatment for systemic envenoming from snakebite [16], but it remains largely unavailable in many parts of rural Sub-Saharan Africa, including Tanzania, where more than 95% of snakebites occur in rural setting [6], leaving many victims at the risk of death or morbidity [16]. Data on the magnitude, management practices and ASV availability are limited in Tanzania.

The present study aimed to determine the community management practices of snakebite cases and availability of ASV products at Meserani Juu village in Monduli District, Tanzania. The study findings obtained highlight on the snakebite envenoming situation in the community, particularly on the management practices and the ASV availability in primary public health facilities in Monduli District. These findings may also be useful in assessing the local and national therapeutic needs in order to improve the clinical management of snakebite envenoming and in planning the provision of snakebite educational programs for the community.

## Materials and methods

### Study design, study area and study period

**Fig 1:**
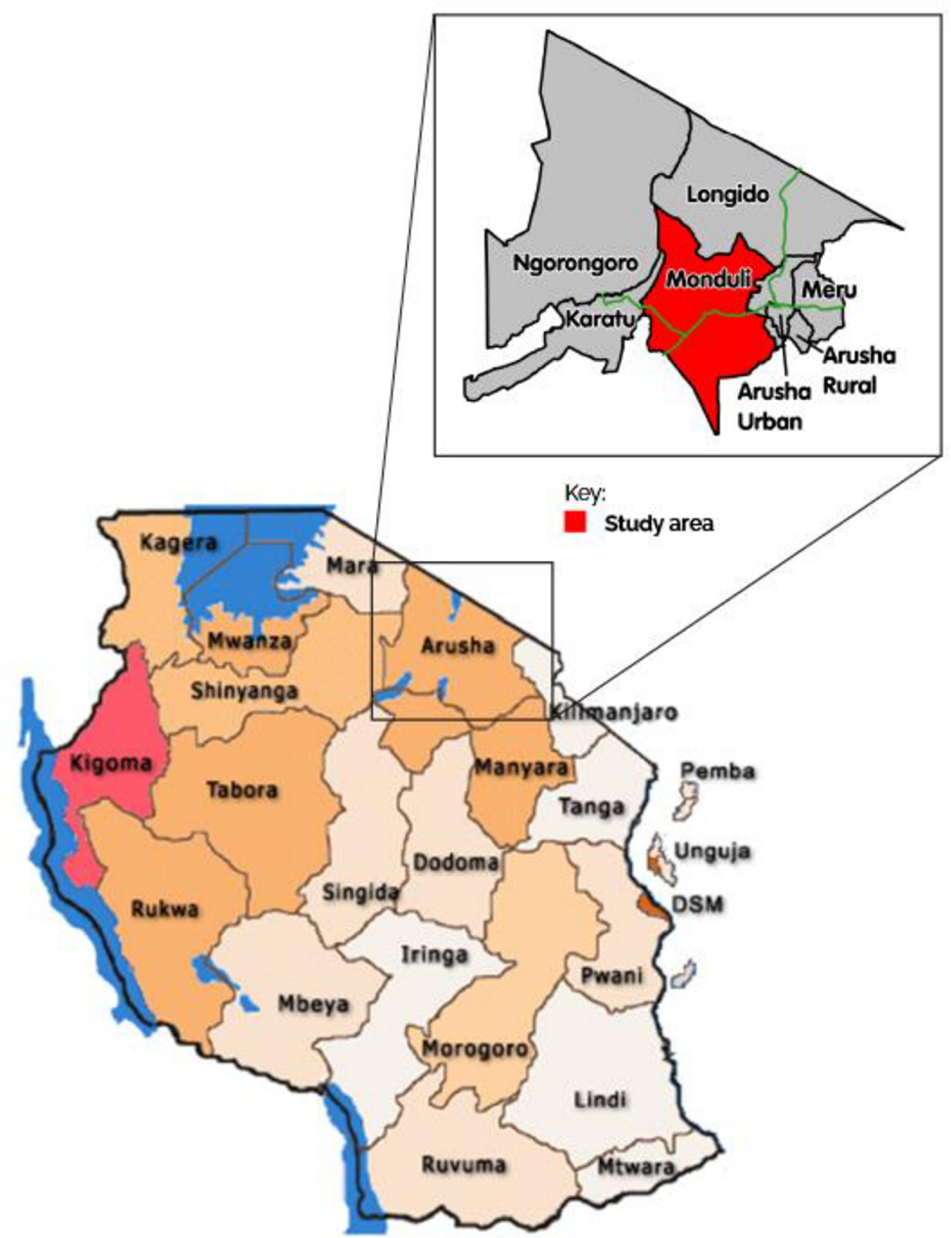
Map of the United Republic of Tanzania showing administrative regions, as well as Monduli district (in red), in Arusha, where the community and the primary public hospitals investigated in this study are located (image adapted from: http://en.wikipedia.org/wiki/Arusha_Region).

This was a cross-sectional study conducted in Meserani Juu village and the primary public health facilities of the Monduli District in Arusha, Tanzania between May and June 2018. Monduli District lies between latitudes 2° and 6° South and longitude 35° to 38° East of Greenwich (Fig 1). Most of the district area is open grassland which is used by Maasai pastoralists as cattle grazing areas and limited agriculture. Livestock production is a type of land use that co-exists with wildlife. According to 2012 Population and housing census, the Meserani villages (Meserani juu and Meserani Chini) have a human population of 11301 [17].The climate of the area is semi-arid with two distinct rainy seasons, short rains in November to December and long rains start in March to May [18] with a mean annual rainfall of about 700 mm. The average maximum temperature is 27°C while the average minimum is 16°C. The selection of the study area was influenced by other findings from previous studies which noted that the area reported many snakebite cases due to its richness in herpetofauna [19, 20]. The snakes identified and suspected of causing envenoming in Monduli district included a black necked spitting cobra (*Naja nigricollis*) and black mamba (*Dendroaspis polylepsis*) of the family Elapidae and the puff adder (*Bitis arietans*) of the family Viperidae as well as other snakes from the families Colubridae and Boidae [20]. The three species (*Naja nigricollis*, *Dendroaspis polylepsis and Bitis arietans*) are enlisted in category 1 in Tanzania by the World Health Organization, corresponding to species of “highly venomous snakes which are common or widespread and cause numerous snakebites, resulting in high levels of morbidity, disability or mortality” [20, 21].

### Study population and data collection

All individuals bitten by snakes in Monduli District in the past five years and other household members living in Monduli were included in the study. A snowball sampling technique was used to identify snakebite victims from households whereby victims were accessed through contact information provided by other respondents. The respondents who met the inclusion criteria were recruited for the study using a purposive sampling technique. A standardized questionnaire was used to collect data concerning snakebites in the past five years through interviews with respondents during a household survey in Meserani Juu village. The questionnaire included information on demographic characteristics of respondents, frequency, season and time of the year of snake encounter, management practices following snakebite, reasons for use of local (traditional) methods for snakebite management and awareness on preventive measures against snake bite. Secondary data from public primary health facilities were extracted using the checklists to capture record on snakebite cases for the past one year and availability of ASV.

### Data analysis

Information from completed questionnaires were coded, entered into spreadsheet and analyzed using a Statistical Package for Social Sciences (SPSS) software version 20. Categorical data were summarized into percentages and presented in tables. Numerical data were summarized into means and standard deviation, and were presented in tables.

### Ethical considerations

Ethical approval for the study was obtained from the joint CUHAS-BMC Research Ethics and Review committee with ethical clearance certificate number 556/2018. Permission to carry out the study was granted by the relevant District and village authority. All study participants signed an informed consent after understanding the purpose and importance of the study prior to data collection. Assent was sought from parents or guardians for participants below 18 years of age. Permission to collect snakebite data and ASV availability information from health facilities records was granted by the respective health facility authorities and the medical record departments of the respective health facilities. The respondents were assured of their right to withdraw from the study at any time they would wish during the interview and of the confidentiality during the whole process.

## Results

### Socio-Demographic characteristics of the respondents

During the study period from May 2018 to June 2018, a total of 214 (67 snakebite victims, 147 other household members) respondents were interviewed. Among the 206 respondents, the mean age was 30.49± 11.4 (Mean ± SD) years in which the minimum and maximum ages were 18 and 76 years respectively. Of the 214 respondents, 121(56.5%) and 93 (43.5%) were males and females respectively. The majority of respondents 133/214 (62.1%) had secondary education, and were farmers 74/214 (34.6%) and pastoralists 64/214 (29.9%). The socio-demographic characteristics of the participants are illustrated in Table 1.

**Table 1:** Socio-demographic characteristics of the respondents (N = 214).

Variable	Frequency (n)	Percent
**Age** * **Age in years:**	30.49± 11.4	
18–38	158	73.8
39–59	44	20.6
≥60	4	1.9
Non response	8	3.7
**Gender:**		
Males	121	56.5
Females	93	43.5
**Head of the family:**		
Father	157	73.4
Mother	41	19.1
Others	16	7.5
**Education level:**		
No education	10	4.7
Primary	62	29
Secondary	133	62.1
Higher education	6	2.8
No response	3	1.4
**Main economic activity:**		
Farmer	64	29.9
Herder	74	34.6
Business	41	19.2
Others	35	16.4

*Age in years in Mean ± SD

### Frequency, season of the year and time of the day of snake encounters

The majority of respondents 183/214 (85.5%) had seen snakes in their village and a half of them 92/183 (50%) had snake encounters more than twice in a year, particularly, during the long rainy season and mostly during day time.

### Description of household snakebite cases

Sixty-seven snakebite cases participated in this study in Meserani juu and more than half were adults. Of all the cases, 31 (53.5%) were females. The majority 58 (89.2%) of the cases were reported to had occurred within the past five years and more than two-fifth 26 (45.6%) of the cases had happened in the afternoon. Only 9 cases were reported to have occurred in the current year of study and more than half of the cases were adults (Table 2).

**Table 2:** The distribution of household cases of snakebite in the Meserani Juu (N = 58).

Variable	Frequency (n)	Percent
**Gender**		
**Male**		
Adults	13	22.4
Children	14	24.1
**Female**		
Adults	19	32.8
Children	12	20.7
**Total**	58	100
**Year of snakebite event**		
Current year	9	15.5
Last year	16	27.6
Past two years	13	22.4
Past three years	9	15.5
Past four years	8	13.8
Past five years	3	5.2
Total	58	100
**Time of the day**		
Morning	5	8.8
Afternoon	26	45.6
Evening	11	19.3
Night	15	26.3
Total	58	100

Children: <17 years; Adults: >18 years

### Management practices after snakebite

All sixty-seven snakebite cases reported to have visited the health facility for treatment after a snakebite. The majority 55/67 (82.1%) of the victims visited a health facility after using some form of local (traditional) treatment as their first aid following snakebite. Only 12/67 (17.9%) of the patients were rushed to the health facilities directly without being given any local first aid and only 3/67 (4.5%) of the victims were sent first to a traditional healer for treatment before visiting a health facility.

The commonest forms of local treatment included the use of tourniquets 13/67 (19.4%), local incision 11/67 (16.4%), and the application of a snake stone (*jiwe la nyoka*) 7/67 (10.4%). Other local remedies used were the ingestion of milk or milk mixed with salt, and drinking of own urine or flushing the eyes of the victim with milk (for the case of venom ophthalmia), placement of coins (mainly fifty or one hundred Tanzanian shillings) or application of unknown herbal preparations (such as *eng’ookoto*) on the wound. Other methods mentioned by respondents included removal of snake fangs, killing of the snake responsible by slicing its head or tail, and washing of wound with milk, and applications of soap, salt, water mixed with salt, and kerosene on the bite site. These data are summarized in Table 3.

**Table 3:** The distribution of various traditional methods used following snakebite event in Meserani Juu (N = 67).

Local first aid measure*	Frequency (n)	Percent
Tourniquet	13	19.4
Local incision	11	16.4
Snake stone	7	10.4
Others	24	35.8
None	12	17.9
**Total**	67	100

*more than one method may be used

Regarding the belief on traditional practices, the majority 80 (40.61%) of the respondents showed a belief in local practices used in the management of snakebites, as they responded positively on the question “Are the local methods helpful?”

With regards to how local methods are helpful, of 76 (35.5%) respondents, 60 (83.3%) said local practices help to prevent the poison from spreading to other part of the body, 9 (11.8%) said it saved life, and few 3 (3.9%) reported that they reduced pain.

### Reasons for the use of local methods for snakebite management

The most frequently reported reasons for the use of local methods were: long distance from sites where snake bite occurred to health facilities 26 (13.7%), followed by failure to believe they will get an appropriate treatment at the facility 22(11.6%), to get first aid prior rush to hospital 16 (8.4%) and lack of money to pay for hospital charges 13(6.8%).

### Awareness on preventive measures against snakebites

A total of 122/214 (57.0%) study participants, responded to the question about preventive measures protecting from snakebites. More than half 82/122 (67.2%) of the respondents knew at least one preventive measure and probably were using preventive measures. The preventive measures reported are such as cleaning the environment round the house, avoid passing around the bush, wearing boots when working in a risky area and planting anti-snake trees/plants around the house [22–24].

Among 208 respondents, more than three quarter 158/208 (75.9%) of respondents were not aware of public health campaigns/programmes addressing snakebite envenomation.

Regarding whether they would like one day to receive that education on snakebite prevention and management, almost all of them 205/214 (97.6%) responded positively with only few 5 (2.4%) with a negative response.

### Snakebite cases reported in various health facilities in Monduli District

There was no documentation of the snakebite cases in any of the primary public health facilities in Monduli District. All snakebite victims who attended primary public health facilities were referred to the Meserani snake park clinic for treatment. A total of 45 snakebite victims attended the Meserani snake park clinic between January 2017 and December 2017. Of all the cases, 21 (46.7%) and 24 (53.3%) were male and female patients respectively. The majority 25 (55.6%) of the victims were children and most of the bites 40 (88.9%) occurred during the rainy season (November-June). These data are presented in Table 4.

**Table 4:** The distribution of the characteristics of snakebite patients recorded at Meserani snake park clinic between January 2017 and December 2017 (N = 45).

Variable	Frequency (n)	Percent
**Gender**		
**Male**		
Adults	9	20.0
Children	12	26.7
**Female**		
Adults	11	24.4
Children	13	28.9
Total	45	100
**Age**		
1–20	27	60.0
21–40	15	33.3
41–60	2	4.4
61–80	1	2.2
Total	45	100
**Time of the day the bite occurred**		
During daytime	17	37.8
At night	22	48.9
Missing	6	13.3
Total	45	100
**Season**		
Dry season ^a^	5	11.1
Rain/ wet season ^b^	40	88.9
Total	45	100.

a = dry season (July–October); b = rain / wet season (November–June)

### Availability of anti-snake venom in public health facilities

Among 35 primary public health facilities in Monduli District, none of them had ASV supply. The Meserani snake park clinic was the only place in the Monduli district with ASV supply where polyvalent ASV (SAMIR polyvalent antiserum/antivenom^TM^) was used to treat some of the snakebite victims.

## Discussion

This study assessed the community management practices of snakebites in Meserani Juu village and the availability of anti-snake venom in the primary public health facilities in Monduli District, Arusha-Tanzania.

In this study, the majority of the respondents were males with an age range between 18–38 years, mostly engaging in farming or pastoralism. This is similar to the findings of a previous study done by Kipanyula and Kimaro, 2015 [20]. Contrary to the previous study carried out in Monduli which recorded that the majority of respondents had a primary education level [20], in the present study, the majority of respondents had a secondary education level. This difference could be explained to the current various efforts made by the government and non-government agencies to educate Maasai society.

Generally, snake bites were found to be a problem in Meserani Juu Monduli district as a high frequency of snake encounters and snakebite cases have been reported. Fifty percent (50%) of the respondents had experienced snake encounter at more than twice. Different results were seen in a study by Nonga and Haruna (2015) again in Meserani chini and Meserani juu in Modnduli District which reported 97% frequency of snake encounters at more than three times [19]. Similarly to the previous studies [19, 20], the majority of snake encounters occurred during the rainy season and during the day (54.9%). This is because the rainy season brings out venomous snakes from their shelters to hunt and breed, thus making this time of the year more hazardous, particularly for agricultural workers and cattle herders.

This study reports sixty-seven (31.5%) snakebite cases out of 214 respondents. A study done in the same place in the year 2015 reported three (7.5%) cases of snakebite out of 40 people interviewed [20]. The big difference is probably due to different sample size and sampling method used. In agreement with a previous study [20], the majority of snakebites happened during the daytime. A total of 45 snakebite cases were recorded at the Meserani snake park clinic retrospectively for the past twelve months (January to December 2017), similar to the cases recorded in the year 2011 (47 cases) but lower than those reported in the year 2012 (56 cases). The nurses at the clinic claimed that the victims that were treated at the clinic not only came from Monduli District, but also from other districts, and even from Manyara regions, mainly from Simanjiro District.

Half of the snakebite victims were females (53%) and more than one-fifth (44.8%) were children. This is similar to the findings from other studies [19, 25]. The reason for this finding could be due to the intense involvement of females in agriculture activities and for the case of children, may be attributed to the natural curiosity and lack of judgment about the danger associated with snake.

With respect to management of snakebite envenomation, it was revealed that more than three quarter of the victims had used some form of local remedy in the management of snakebite at Meserani Juu. This finding is in agreement with the findings of the study done in Nigeria [15] and KwaZulu-Natal South Africa [26], whereby the use of some form of traditional methods prior to hospital admission was found in 81% and 90% of the victims respectively. Moreover, the findings of this study revealed the application of tourniquet just above or below the bite area to be the most common method used, although its use is controversial [15, 27, 28] due to the contradicting issues that tourniquet will prevent the venom from spreading to other parts of the body but may cause venom to stay concentrated near the bite site causing the rapid destruction of body cells; however allowing it to spread will dilute the toxin and reduce tissue damage. This finding about tourniquet use is similar to the findings from previous studies, even though they recorded a much higher frequency of tourniquets application of (63.1%), (74%), and (83%) respectively [15, 19, 26]. The lower rate in the present study may be due to the presence in the area of Meserani Snake Park and its clinic, a popular tourist attraction and a clinic providing free antivenoms for snakebite victims.

Some of the local methods to manage snakebites reported in the present study were also reported in previous studies [10, 14, 19, 25, 26, 29, 30]. However, according to study findings, the use of traditional methods was found to be associated with an increased risk of death and disability [12, 15, 26, 29].

The most frequently reported reasons for the use of traditional methods in the present study were the long distance from the bite site to a health facility (13.7%), followed by bad beliefs about treatment provided in hospitals (11.6%), and the need for first aid prior to rushing to hospital (8.4%). The findings in this study are in line with the findings from previous studies [9, 11–14]. Furthermore, the current study revealed that only a few (5.7%) of the victims were sent first to a traditional healer following snakebite before going to a health facility while a study done in Zimbabwe estimated that 16% of patients initially consulted traditional healers following a snakebite [31]. Results seen in a study done in Kedougou Eastern Senegal showed that 76.8% of snakebite victims first consulted traditional healers [13].The variations in the study results may be due to the choice of the place of the survey, differences in socio-demographic characteristics most probably, frequency of bites and economic activities. Most probably, a population using traditional medicine for any other ailment will also use it for snakebite.

The current study demonstrated that ASV,—the only specific treatment available for systemic envenomation, was not available in all primary public health facilities in Monduli District. The district had a total of 34 dispensaries, 2 health centers, one district military hospital, and a district hospital. This was a novel finding and confirms the results of a previous study in Kenya which found polyvalent antivenom to be rarely present in medical units in the area studied [29].

The only ASV available at Meserani snake park clinic (a private clinic run by nurse attendants) was the polyvalent antivenom (SAIMR polyvalent snake aniserum/antivenom^TM^). The ASV was provided free of charge at the clinic though the current cost of each 10 ml vial is around 400 US dollars, equivalent to 910,000 Tanzanian shillings. It was also found that for the majority of snakebite victims, only a single vial was enough to improve the symptoms of envenomation. However, for severe envenomation cases, a patient may require two to eight ASV vials. This finding clearly shows that the availability of an effective, safe and affordable antivenom remains a problem and this leaves the majority of snakebite victims living in developing countries without access to a proper treatment [16, 32].

### Limitation of the study

One of the major limitations of this study was a sampling error due to the failure to achieve a minimum sample size, however more than 97% of the required sample was attained. In addition, since the study findings were based on the reports by participants there is a potential for recall bias, however a period of past five years was used to minimize recall bias.

## Conclusion

Generally, snakebite was found to be a problem in Meserani Juu village, affecting mostly pastoralists and crop farmers. More than three quarters of the snakebite victims used some form of local practices in the management of snakebites, of which most are of unknown efficacy and safety. Furthermore, antivenoms were found to be out of stock in all primary public health facilities found in Monduli District despite the area being a habitat for different kinds of venomous snakes. Based on these findings, we recommend further studies covering larger areas and different parts of the country in order to get a clear picture of this neglected tropical disease. Finally, the government, traditional healers, and non-government organization should collaborate so as to improve the health services, antivenom availability at a regional level, especially in the remote rural areas and plan the implementation of education and preventive programs to the rural communities to increase awareness on snakebite envenoming and its management.

## Supporting information

S1 File Questions to participants. (DOCX)
