# Supplementary material for: Assessment of snakebite management practices at Meserani Juu in Monduli District, Northern Tanzania
Source: PLoS One. 2022 Dec 22;17(12):e0278940. doi: 10.1371/journal.pone.0278940 (PMC9778626; doi:10.1371/journal.pone.0278940)
Supplement: S1 File — (DOCX) [file pone.0278940.s001.docx]

| **Qn.#** | **PART A: SOCIO DEMOGRAPHIC AND SOME BASIC INFORMATIONS** |  |
| --- | --- | --- |
|  | Age of the respondent (in years) __________ |  |
|  | Gender of the respondent   1. Male 2. Female |  |
|  | Who is the head of the family in this household?   1. Farther 2. Mother 3. Others (Specify)_____________________________ |  |
|  | What is the level of your education?   1. No formal education 2. Primary 3. Secondary 4. Higher education |  |
|  | What is your main economic activity?   1. Crop Farmer 2. Herder 3. Business 4. Other (specify) |  |
|  | **PART B: SNAKE ENCOUNTERS AND SNAKEBITE INFORMATIONS** |  |
|  | Have you ever seen a snake?   1. Yes 2. No   *If the answer is yes continue with the next questions* |  |
|  | How often have you encountered with the snake?   1. Less than one in a year 2. Once in a year 3. More than twice in a year |  |
|  | At what period of the year?   1. During long rainy season 2. During short rainy season 3. During dry season |  |
|  | At what time did you encounter with a snake?   1. In the morning 2. At noon 3. In the evening 4. At night |  |
|  | Have you ever been bitten by a snake? / Did any household member have been bitten by snake?   1. Yes 2. No   *If the answer is Yes continue with the next questions*  *If the answer is No go to question number16* |  |
|  | When?   1. In the past 5 years 2. In the past 4 years 3. In the past 3 years 4. In the past 2 years 5. In last year 6. This year 7. Long time ago (specify)_________________________ |  |
|  | Under what circumstances did you get bitten by snake?   1. While walking 2. While sleeping 3. While working(specify)_____________________ |  |
|  | At what time of the day?   1. Morning 2. Afternoon 3. Evening 4. Night |  |
|  | **PART C: SNAKEBITE MANAGMENTS PRACTICES** |  |
|  | What did you do immediately after being bitten by a snake?  Please explain______________________________________________  ____________________________________________________ |  |
|  | Do you know any local (traditional) practices used in the management of snakebite in your area?   1. Yes 2. No   If Yes,  Mention them________________________________________  ____________________________________________________ |  |
|  | Are those methods helpful?   1. Yes 2. No 3. Not sure   If Yes,  Explain______________________________________________  ____________________________________________________ |  |
|  | Why people still practice or use those traditional methods in the management of snakebite?  ____________________________________________________ |  |
|  | Do you know any traditional healer in this village who treat snakebite victim?   1. Yes 2. No |  |
|  | Have you ever gone / did you ever sent a snakebite victim(s) to a traditional healer?   1. Yes 2. N o |  |
|  | What reasons made you decide to visit a tradition healer?  ____________________________________________________ |  |
|  | Why people do not decide to first visit a health facility following snakebite?  Give your opinions/reasons______________________________  ____________________________________________________ |  |
|  | Do you know any other community member in the village who has been bitten by a snake?   1. Yes 2. No |  |
|  | What preventive measures do you know or practices to protect from snakebite?  ____________________________________________________  ____________________________________________________ |  |
|  | Have you ever heard of any public health campaigns or programs which address and provide free education to community concerning snakebite envenomation?   1. Yes 2. No   If Yes, Explain______________________________________________ |  |
|  | Would you like to receive any community education program(s) or snakebite prevention campaign(s) in your area?   1. Yes 2. No |  |
